# Preclinical Study of DCD and Normothermic Perfusion for Visceral Transplantation

**DOI:** 10.3389/ti.2023.11518

**Published:** 2023-09-08

**Authors:** Javier Serradilla, Ane Miren Andrés Moreno, Paloma Talayero, Paula Burgos, Mariana Machuca, Onys Camps Ortega, María Teresa Vallejo, Francisco Javier Rubio Bolívar, Alba Bueno, Alba Sánchez, Cristina Zambrano, Carlos Andrés De la Torre Ramos, Olaia Rodríguez, Carlota Largo, Pilar Serrano, Gerardo Prieto Bozano, Esther Ramos, Manuel López Santamaría, Pablo Stringa, Francisco Hernández

**Affiliations:** ^1^ Department of Pediatric Surgery, La Paz University Hospital, Madrid, Spain; ^2^ Transplant Research Group, Institute for Health Research IdiPaz, Madrid, Spain; ^3^ Department of Immunology, University Hospital 12 de Octubre, Madrid, Spain; ^4^ Department of Cardiovascular Surgery, La Paz University Hospital, Madrid, Spain; ^5^ Special Pathology Laboratory, Faculty of Veterinary Sciences, National University of La Plata, La Plata, Argentina; ^6^ Molecular Imaging and Immunohistochemistry Laboratory, Institute for Health Research IdiPaz, Madrid, Spain; ^7^ Department of Experimental Surgery, La Paz University Hospital, Madrid, Spain; ^8^ Department of Biochemistry, La Paz University Hospital, IdiPaz, Madrid, Spain; ^9^ Intestinal Rehabilitation and Transplantation Unit, La Paz University Hospital, Madrid, Spain; ^10^ Institute for Immunological and Pathophysiological Studies (IIFP), National University of La Plata, National Council of Scientific and Technical Research (CONICET), La Plata, Argentina

**Keywords:** donation after circulatory death, normothermic perfusion, intestinal transplantation, experimental DCD feasibility, intestinal graft viability

## Abstract

Considering recent clinical and experimental evidence, expectations for using DCD-derived intestines have increased considerably. However, more knowledge about DCD procedure and long-term results after intestinal transplantation (ITx) is needed. We aimed to describe in detail a DCD procedure for ITx using normothermic regional perfusion (NRP) in a preclinical model. Small bowel was obtained from pigs donors after 1 h of NRP and transplanted to the recipients. Graft Intestinal samples were obtained during the procedure and after transplantation. Ischemia-reperfusion injury (Park-Chiu score), graft rejection and transplanted intestines absorptive function were evaluated. Seven of 8 DCD procedures with NRP and ITx were successful (87.5%), with a good graft reperfusion and an excellent recovery of the recipient. The architecture of grafts was well conserved during NRP. After an initial damage of Park-chiu score of 4, all grafts recovered from ischemia-reperfusion, with no or very subtle alterations 2 days after ITx. Most recipients (71.5%) did not show signs of rejection. Only two cases demonstrated histologic signs of mild rejection 7 days after ITx. Interestingly intestinal grafts showed good absorptive capacity. The study’s results support the viability of intestinal grafts from DCD using NRP, contributing more evidence for the use of DCD for ITx.

## Graphical Abstract

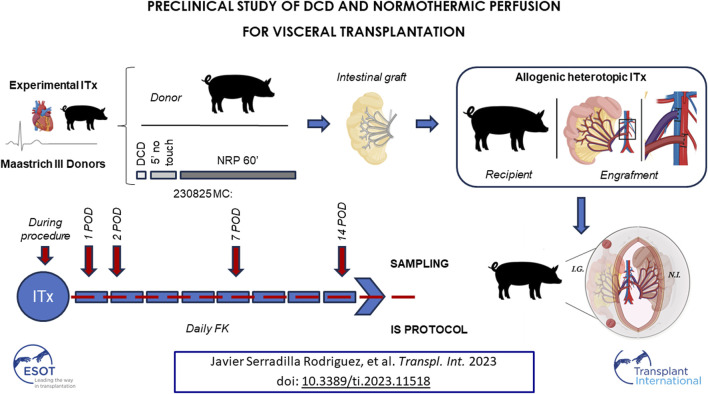

## Introduction

The universal shortage of organs has prompted the growing use of donation after circulatory death (DCDs). Kidneys, liver, lungs, pancreas, and, more recently, the heart from DCDs have been successfully employed for transplantation in many centers worldwide [1, 2]. Most of the pitfalls and concerns regarding DCD have been addressed by strict donor selection [3], improvements in normothermic regional perfusion (NRP) [4] and *ex vivo* machine perfusion devices [5]. Although the true influence of routine DCD use is difficult to assess, this form of organ donation has been recognized to increase the pool of donors [6].

Intestinal transplantation (ITx) is used in selected cases of irreversible intestinal failure, as it has clinical and economic advantages over prolonged use of parenteral nutrition [7]. However, the use of DCD as a source of intestinal grafts has been denied through years due to concerns about ischemic susceptibility [8]. The experimental studies that support this point of view are limited and involve heterogeneous methodologies and currently, it is contrasted with updated experimental evidence that shows DCD as promising in the field of intestinal and multivisceral transplantation [9, 10]. In addition, the clinical evidence supporting the use of these grafts in the clinical setting is promising but scant [11, 12]. Recently, our work group has reported the first case of pediatric DCD and multivisceral transplantation with a good outcome to date [13].

Currently, the search for strategies that increase the availability of solid organs for transplantation remains a challenge for the medical-scientific community [14]. Among patients awaiting solid organ transplantation, ITx candidates show longer waiting periods and high morbidity and mortality rates. This is especially relevant for pediatric patients, who also suffer from other secondary complications, such as lack of growth, increased family dependency and lack of social and academic development.

Therefore, further evaluation of the potential use of DCD intestinal grafts is warranted. Taking this background into account, this study aimed to evaluate the feasibility of DCD intestinal grafts in a preclinical model of ITx.

## Materials and Methods

### Animal Use and Care

Sixteen Large White pigs (25.5 ± 2.5 kg) were used for ITx (eight donors, eight recipients). They were all female, non-related, outbred, and aged 8–10 weeks to ensure uniformity in veterinary management. Prior to use, all animals underwent strict veterinary control that determined their good health and condition. The protocol was approved by the Animal Welfare Ethics Committee (PROEX 58.7/20) and complied with the EU and Spanish Directives on experimental animals (63/2010 EU, RD 53/2013). The entire donation process under DCD conditions was strictly controlled by the veterinary staff of our institution.

### Circulatory Death Induction, NRP, and Donor Surgery

The animals were starved for 24 h before surgery. Premedication was performed directly in the box (12 mg/kg of ketamine intramuscularly + 0.5 mg/kg of midazolam). With the animals under inhalational anesthesia (sevoflurane, 4%), we performed catheterization of the ear veins and arteries and endotracheal intubation, which was followed by a propofol (11 mg/kg) and fentanyl (5.4 μg/kg/h) infusion. A 14-gauge angiocath was inserted in the fifth intercostal space in the mid-axillary line. Withdrawal of life support therapy was performed, and a bolus of unfractionated heparin (300 IU/kg) was injected through a central venous catheter. Manual air thoracic insufflation (100 mL/2′) was performed to induce progressive tension pneumothorax and subsequent lethal cardiovascular collapse. After 200 ± 50 mL, a persistent decline in oxygen saturation level was observed. Functional warm ischemic time (fWIT) was initiated when oxygen saturation was sustained at <80% and/or mean blood pressure was <50 mmHg, as established by the Spanish legislation for DCD in clinical practice [15]. Death was declared after cardiac arrest and a 5 min “no-touch period.”

A rapid laparotomy was performed, and the aorta and inferior vena cava were cannulated (14/16 French [Fr] and 20/22/24 Fr, respectively; DLP™ Medtronic, Minneapolis, MN, United States). Thoracotomy was performed, and the descending thoracic aorta was cross-clamped to prevent perfusion of the brain and coronary arteries. Subsequently, NRP was established using a compact custom closed extracorporeal circulation circuit (Rotaflow™ RF-32 centrifugal pump; Maquet, Hirrlingen, Germany). The detailed procedure for NRP control and determinations during this 1 h period are provided as Supplementary Material.

During NRP, the small bowel was prepared for procurement. After 1 h of NRP, the circuit was stopped, and the same cannulas were used for cold perfusion with cold-preservation solution (Celsior^R^, IGL). The graft comprised the small bowel from the duodenum to the terminal ileum, with a vascular pedicle formed by the superior mesenteric artery and portal vein. During this cold ischemia time (CIT), the graft was kept in cold storage using the same solution until engraftment.

### Recipient Procedure

Heterotopic ITx was performed. The native bowel was shifted to the left side of the abdomen, and the infrarenal aorta and cava were exposed. Portocaval anastomosis, followed by anastomosis between the superior mesenteric artery and aorta, was completed, and reperfusion of the bowel was established. The proximal end of the bowel remained closed, and a terminal ileostomy was performed for graft sampling. A gastrostomy was performed to facilitate daily administration of immunosuppressive drugs, antibiotics and analgesics during the post-transplant monitoring of the animals.

### Recipient and Graft Clinical Monitoring

After ITx, the recipients were individually isolated with controlled feed and water *ad libitum*. The animals’ welfare was supervised twice a day. Analgesia was provided with a fentanyl patch every 3 days for 1 week and with ibuprofen (10 mg/kg/12 h for 7 days, by tube). Cefixime 20 mg/kg was administered every 12 h. Tacrolimus was administered daily, and the doses (0.2–1 mg/kg/24 h) were adjusted according to serum levels. All drugs were administered through gastrostomy.

Ileoscopy was performed through the ostomy using a 9 mm pediatric endoscope on postoperative days (PODs) 1, 2, 7, and 14. Four biopsies were obtained during the examination. The animals were sacrificed and sampled on POD 14 by intravenous injection of 5M KCl under general anesthesia.

### Sample Collection

Graft samples were obtained at 30′ and 60′ after NRP initiation. An additional sample was obtained after flushing with the cold-preservation solution. Three intraoperative intestinal graft samples were obtained as follows: pre-reperfusion, immediately after reperfusion, and 60′ later. Samples were always taken from the ileum for uniformity. Additional intestinal samples were obtained on PODs 1, 2, 7, and 14 through the ileostomy. Blood samples were obtained at 5′, 30′, and 60′ after the beginning of NRP (donor) and on PODs 1, 2, 7, and 14 (recipient). Baseline samples were collected from each animal.

### Histopathological Analysis

Histological analysis was performed on 5 μm hematoxylin–eosin-stained tissue sections. Ischemia-reperfusion injury (IRI) was evaluated using the Park-Chiu score (PCS), whereas the Wu score was used to evaluate rejection.

### RNA Isolation and Gene Expression Assessment

Mucosal intestinal biopsy specimens were immediately submerged in RNAlater solution (Invitrogen, Waltham, MA, United States) and stored at 4°C for 24 h and then at −80°C. RNA was isolated using the RNeasy Mini Kit (Qiagen, Hilden, Germany) and retrotranscribed into cDNA using a High-Capacity cDNA Reverse Transcription Kit (Applied Biosystems, Waltham, MA, United States).

Expressions of genes for tight junction protein 1 (TJP1), epithelial cell adhesion molecule (EpCAM), mucine 2 (MUC2), lysozyme (LYZ), interleukin (IL)-6, and tumor necrosis factor alpha (TNF-α) were measured using predesigned TaqMan™ Gene Expression assays (Ss03373514_m1, Ss03384752_u1, Ss03377386_u1, Ss03394856_m1, Ss03384604_u1, Ss03391318_g1) with TaqMan™ Fast Advanced Master Mix on a 7500 Fast Real-Time PCR System (Applied Biosystems). Threshold cycle (Ct) scores were calculated as the mean of the duplicates and normalized against the Ct scores of the endogenous control GAPDH (Ss03375629_u1). Relative expression was determined as 2^−ΔCt^, where ΔCt = Ct gene of interest–Ct endogenous control [16]. 2^−ΔCt^ values were normalized by logarithmic transformation, and fold-change values were calculated using the median value of native intestines as a reference.

### Functional Evaluation of Transplanted Intestines

Citrulline levels were measured sequentially (baseline, PODs 1, 2, 7, and 14) as a biomarker of small bowel mass and function. The absorptive function of the grafts was evaluated on POD 14, as previously reported [17]. A solution containing glucose (2 g/kg) was intraluminally administered to the transplanted intestines. The glucose level of peripheral blood (tongue) and draining veins from both the intestinal graft and native small bowel was measured (Accu-Chek blood glucose meter, Roche) immediately before and 15′, 30′, and 60′ after glucose administration to verify whether the increase was due exclusively to the absorption of the solution and not to a pre-existing blood glucose level from its native intestine.

### Statistical Analysis

Differences in gene expression values between grafts and native bowels were assessed using the unpaired t-test (with the Welch correction for significantly different variances). In contrast, the paired t-test was used to compare the graft samples at different time points. Spearman rho and linear regression values were calculated for correlation analysis between gene expression values and ischemia times (fWIT or CIT). Citrulline levels were compared among the different time-points using the paired t-test. Statistical significance was set at *p* < 0.05, and GraphPad Prism (version 8.0.2) software was used for all the tests.

## Results

### Experimental DCD Feasibility, Transplanted Recipient Outcome, and Graft Viability

**FIGURE 1:**
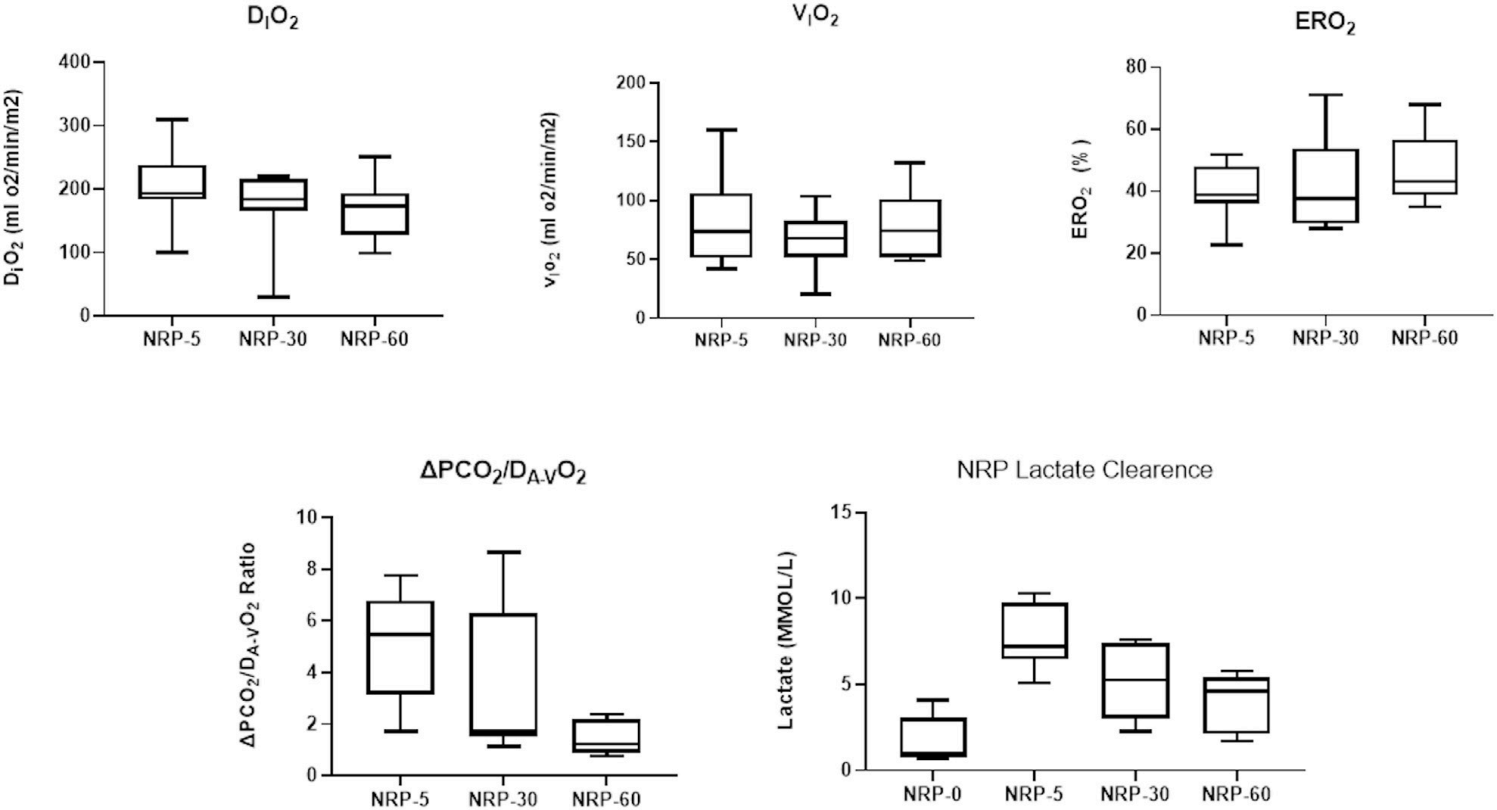
Monitoring during NRP: Oxygen-derived parameters [indexed O_2_ delivery (D_i_O_2_), indexed O_2_ consumption (V_i_O_2_), O_2_ extraction (ERO_2_)], carbon dioxide-derived parameters (ΔpCO_2_/DA-VO_2_ Ratio), and lactate clearance were evaluated at different timepoints of NRP during donor procedure. NRP, normothermic regional perfusion.

Pneumothorax-induced cardiac arrest within <5′, and fWIT was 20′ ± 5′. NRP achieved good reperfusion of the abdominal organs and lactate clearance in all cases. The laboratory variables analyzed are summarized in Table 1 and Figure 1. After 1 h of NRP, 8/8 intestines were successfully obtained from DCD since all eight showed a good macroscopic appearance during graft dissection and intravascular washing. After 147.8′ ± 12′ of CIT, the grafts were implanted, achieving good graft reperfusion in 7/8 procedures (87.5%). In one case, the transplanted small bowel was poorly reperfused because of venous stenosis; hence, this animal was excluded. The remaining seven recipients recovered adequately. One animal with a gastrostomy leak required an anticipated endpoint on POD 10, while the remaining six reached the scheduled POD 14.

**TABLE 1:** Analytical monitoring during NRP.

		NRP		
	Baseline	5 min	30 min	60 min
AST (UI/L)	31.6 ± 11.94	38 ± 20.43	39.5 ± 15.45	49.16 ± 21.62
ALT (UI/L)	49.66 ± 14.88	36.16 ± 8.43	32.66 ± 9.97	25.83 ± 5.20
Urea (mg/dL)	24.5 ± 5.28	20.4 ± 3.55	22.66 ± 4.02	22 ± 3.95
Creatinine (mg/dL)	1.14 ± 0.14	1 ± 0.27	0.98 ± 0.27	0.83

Aspartate aminotransferase (AST), alanine transaminase (ALT), urea and creatinine were measured during DCD and NRP.

### Endoscopic Follow-Up of the Graft

The distal 40–50 cm of the graft was explored. All cases showed a mucosa with normal appearance, coloration and vascularization, without erosions, ulcers or bleeding, with a well-preserved villous pattern.

### IRI and Graft Rejection

**FIGURE 2:**
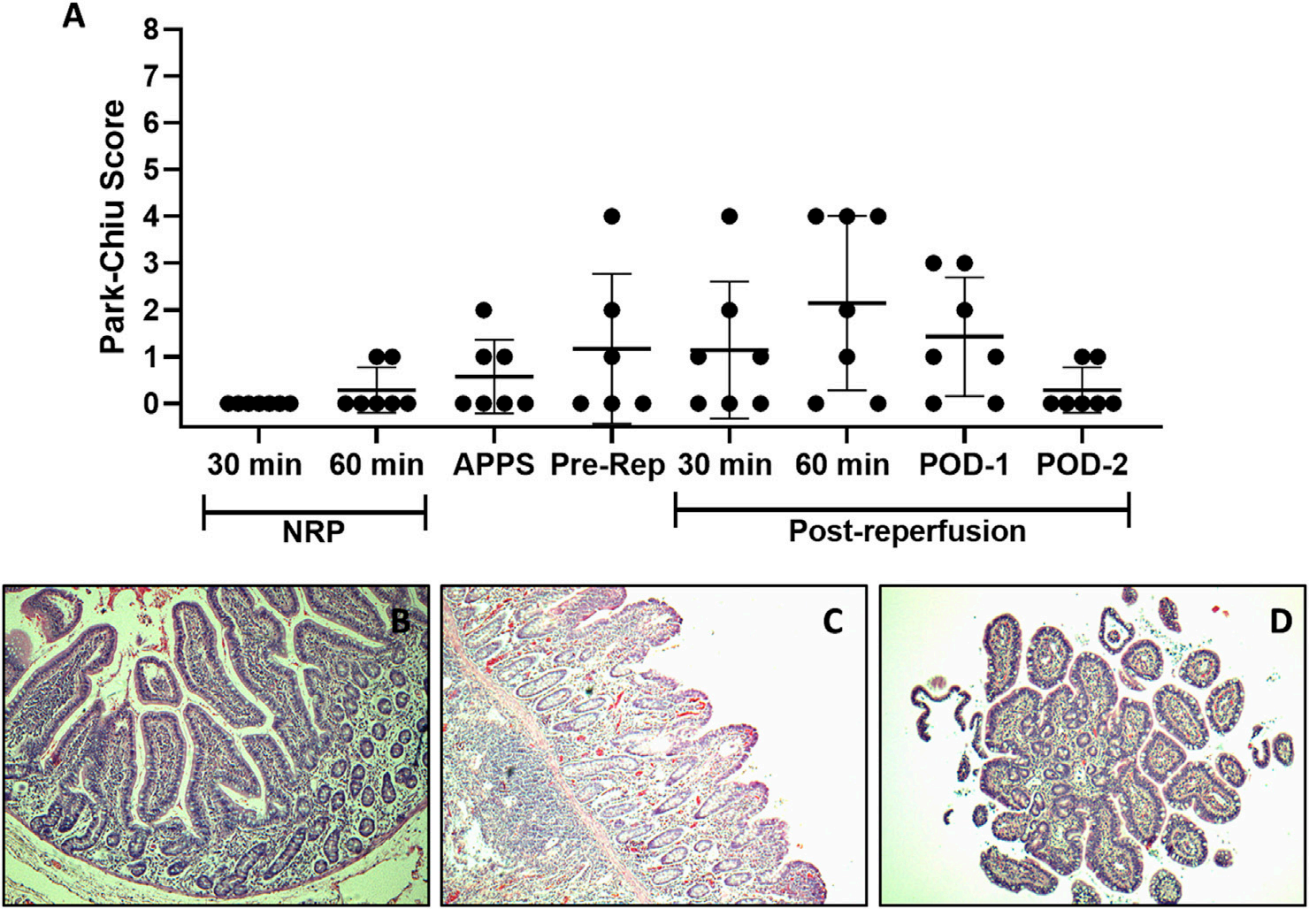
Intestinal IRI analysis. Histological damage at different stages of the ITx procedure with DCD and NRP was studied using the PCS. Each point depicts a simple sample evaluation. **(A)** Microscopic representative images of an intestine after 60 min of NRP **(B)**, 60 min, **(C)** and 2 days after graft reperfusion **(D)** (H/E-stained samples, ×10). APPS, after perfusion with preservation solution; ITx, intestinal transplantation; DCD, donation after circulatory death; NRP, normothermic regional perfusion; H/E, hematoxylin–eosin.

As shown in Figures 2A–D, in both NRP-30′ and NRP-60′ samples, the small bowel showed a well-preserved architecture. However, two NRP-60′ samples showed edema at the villus tip (PCS 1). As expected, the highest IRI was observed after 1 h of graft reperfusion, with denuded villi appearing in 3/7 evaluated samples. Interestingly, all grafts recovered 48 h after transplantation.

**FIGURE 3:**
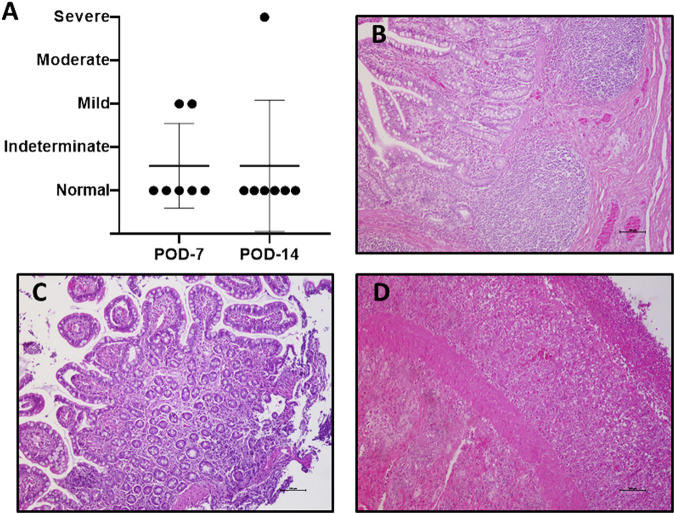
Graft rejection. Histological study in search of acute cellular rejection was performed using the Wu scheme **(A)**. Most of the evaluated samples were normal **(B)**. A few cases present microscopic alterations, consistent with mild rejection, **(C)** and only one case of severe rejection on POD 14 was observed **(D)**. (H/E-stained samples, ×10). POD, postoperative day; H/E, hematoxylin–eosin.

Mild rejection appeared on POD 7 in 2/7 (28.5%) cases; one showed full recovery, and the other showed severe acute cellular rejection on POD 14 (1/7, 14.2%). The remaining 5/7 (71.4%) recipients showed no signs of rejection at any point (Figures 3A–D).

### Gene Expression Analysis

**FIGURE 4:**
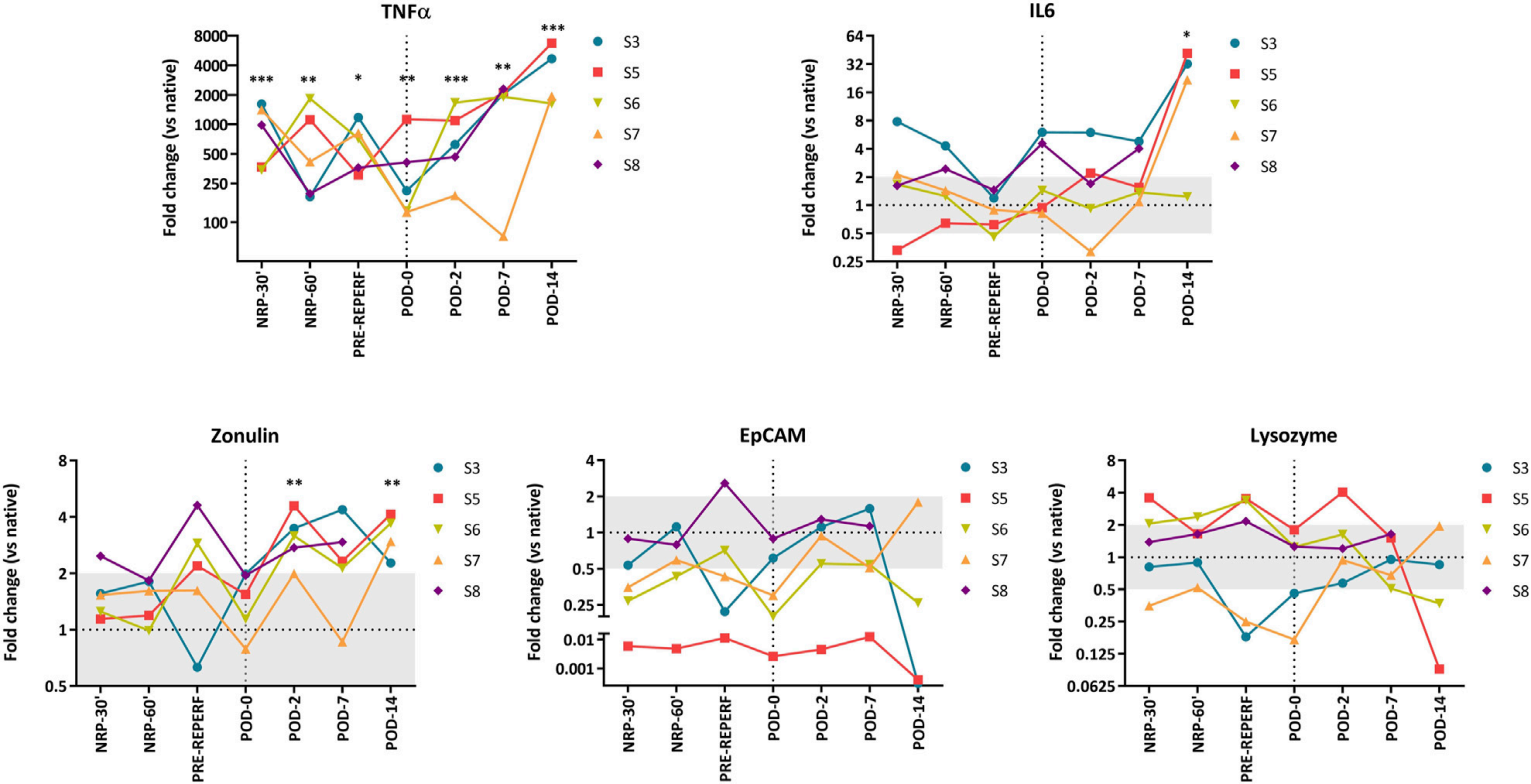
Fold change gene expression values. Each line represents values from a single animal. Comparison between grafts and native intestines was made using an unpaired t-test. Significant *p*-values are plotted as follows: *: *p* < 0.05, **: *p* < 0.01, ***: *p* < 0.001.

Relative expressions of integrin EpCAM, TJP1 (zonulin), LYZ, MUC2, the proinflammatory cytokines IL-6, and TNF-α were assessed by quantitative polymerase chain reaction in serial samples (NRP-30′, NRP-60′, pre-reperfusion, and PODs 0, 2, 7, and 14) of five animals and three additional samples of independent native bowels (Figure 4 and Supplementary Figure S1). MUC2 was excluded because it was undetectable in most samples. NRP-30′ samples showed differences in the molecular graft signature of native intestines (Figure 4) and a dramatic 350–1600-fold increase in TNF-α levels (*p* < 0.001). IL-6 levels also increased 2–8-fold in four of the five animals (*p* = 0.17). Among genes related to epithelial integrity, zonulin showed an almost significant upregulation (*p* = 0.06), while EpCAM appeared to be downregulated in all samples (not significant because of a high dispersion). Generally, LYZ expressions were quite similar to those in the native intestines.

Next, we evaluated the possible correlation between fWIT, CIT, NRP times and gene expression values, were no significant association was observed (Supplementary Figure S1).

In samples taken 1 h after reperfusion, gene expression values seemed to normalize at the end of the transplantation procedure (POD 0), except TNF-α, which remained high (*p* = 0.002). Interestingly, no relevant changes in gene expression were observed when compared to the pre-reperfusion samples, and IL-6 expression levels showed only a slight increase (*p* = 0.055).

During the post-transplantation period, TNF-α expression persisted above the native intestinal levels, differing significantly at every time point studied (POD 2, *p* < 0.001; POD 7, *p* = 0.002; POD 14, *p* < 0.001). Additionally, upregulation of this gene became evident on POD 7, becoming significant on POD 14 (*p* = 0.003). The maximum expression values were also observed at this point, with 1600–6700-fold changes. A similar, albeit less prominent, dynamic was observed for IL-6, which showed stable expression until POD 7, followed by an abrupt increase (except in one animal) at the endpoint (*p* = 0.037 versus controls).

The epithelial-related genes EpCAM and zonulin showed significant upregulation on POD 2 compared to POD 0 samples (*p* = 0.008 and *p* = 0.005, respectively). On POD 7, these differences persisted (*p* = 0.019 for both) and remained until POD 14 for zonulin (*p* = 0.042). Moreover, zonulin expression remained significantly higher than that in native bowels throughout the post-transplantation period (POD 2: *p* = 0.003; POD 7: *p* = 0.055; POD 14: *p* = 0.004). LYZ expression varied among the animals, and it seemed to be lower than that in native intestines; however, it was not significantly different from the POD 0 samples.

### Intestinal Graft Functional Evaluation

**FIGURE 5:**
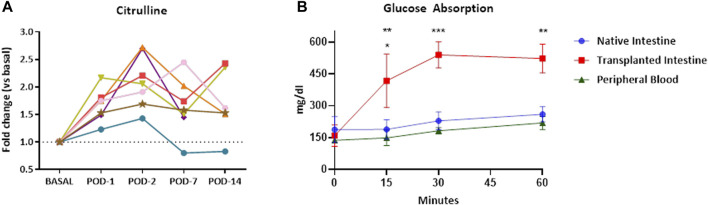
Graft functional evaluation. Citrulline plasma levels increase after ITx. Each line represents values from a single recipient **(A)**. The glucose absorption test of the transplanted intestines from DCD procedures was performed by introducing a glucose solution into them. The transplanted intestine was observed to have a good absorptive capacity. The glucose level increases peripherally and in native intestines as it is distributed through the body **(B)**. Results are presented as the mean ± SEM. Significant *p*-values are plotted as follows: *: *p* < 0.05, **: *p* < 0.01, ***: *p* < 0.001. ITx, intestinal transplantation; DCD, donation after circulatory death; SEM, standard error of the mean.

Citrulline blood levels increased significantly in all cases in the first 2 days post-ITx (*p* = 0.0003). Despite differences between the animals after this point, only animal 3 showed levels below the baseline, which corresponded to severe acute cellular rejection in the histological examination (Figure 5A). Graft-absorption capacity on POD 14 was evident 15′ after glucose administration, reaching maximum glucose levels at 30′. Elevated graft blood glucose levels were maintained throughout the study period (Figure 5B). In addition, blood samples from the peripheral circulation and native small bowel showed a slow and gradual increase in glucose levels, confirming that the increase in the glucose level is due to the graft’s glucose absorption and its distribution to the general circulation.

## Discussion

Intestinal grafts from DCD are universally considered non-viable for transplantation, mainly because of the vulnerability of the intestine to IRI [18] and the relationship between graft quality and loss of barrier function. However, recent improvements in DCD and ITx have opened a new window of opportunity to increase the limited pool of donors. Our experimental study aimed to further validate the potential of DCD grafts for clinical ITx, enhancing knowledge of DCD and visceral transplantation and increasing the scientific evidence that supports the use of this type of donor. Under experimental settings, fWIT, a critical factor for graft viability, was similar to that reported in other studies [19]. The CIT was shorter than the usual time in clinical settings, only for logistic reasons, and was far from the established 9 h limit for acceptance [12].

We used large white pigs for this model. Numerous studies have confirmed this species to be the most suitable research model for human ITx because of its similarity in size, physiology, immunology, organ development, and function [20]. Our immunosuppressive treatment scheme with daily tacrolimus has already demonstrate its efficacy [21, 22].

Performing a heterotopic transplant allows the study of the viability and function of the grafts without affecting the digestive function of the animals while avoiding the possible surgical complications that could arise from an orthotopic transplant. In addition, it allows possible pathological events that may occur in the graft to be tolerated much better [23]. Therefore, in our experience this modality allows the animals to maintain a good general health status without affecting the study objectives.

Despite the importance of intestinal IRI, the effect of NRP has been scarcely evaluated. Guo et al. demonstrated that intestinal conditioning with 1 h of extracorporeal membrane oxygenation (ECMO) improved the energy status and graft viability in a pig model [24]. Nevertheless, these improvements were compromised gradually as the extracorporeal support used by Hamed et al. [25] demonstrated the viability and function of the intestine from DCD in an experimental model with *ex vivo* normothermic perfusion for 4 h. Unfortunately none of these studies included transplantation as proof of viability. Guo et al. [26] published another well-designed study overcoming this limitation. They compared graft viability and function after 7 days in three experimental groups: living donor, DCD (rapid technique), and DCD with ECMO. Cell apoptosis and endotoxin levels were lower and intestinal absorptive function was significantly better in the ECMO group than in the DCD group. They concluded that ECMO exerted a protective effect on IRI, probably by reducing caspase-3 protein expression and apoptosis. These results are consistent with our findings since the mucosal function and structure were almost normal for 7 days, and only minor histological changes appeared after 14 days, which might be attributed to incipient rejection. Moreover, our gene expression analysis demonstrated that TNF-α, zonulin, pCAM, and LYZ expressions at NRP-60′ were similar to those before reperfusion, indicating graft integrity during NRP and adding more valuable information regarding the recovery effect of ECMO support after DCD. Thus, our findings and the previous literature suggest that NRP transforms the deleterious influence of IRI inherent to DCD into an “ischemic preconditioning” phenomenon to improve intestinal graft viability. However, Softeland et al. [27] performed a comparative study between species on the development of intestinal epithelial lesions and observed that these lesions develop more slowly in pigs. Although it is a study of non-transplanted intestinal samples, this fact should be considered when interpreting the findings.

Additionally, the molecular study showed a >250-fold-increase in TNF-α expression at the beginning of the procedure. Hypoxia could upregulate the TNF-α pathway through NF-κB activation after increasing mitochondrial reactive oxygen species in innate immune cells [28]. In addition, intestinal epithelial cells (IECs) respond to low-oxygen conditions by secreting TNF-α, which is derived from epithelial barrier disruption [29, 30]. One of the mechanisms by which TNF-α directly increases permeability is through the induction of occludin endocytosis via myosin light chain kinase activation [31]. Although the PCS for most samples showed no or mild damage in the intestinal mucosa and no clinical manifestations of barrier disruption, the molecular profile of epithelial-related genes demonstrated certain changes. Thus, a significant increase in EpCAM (an IEC integrin), and especially zonulin (a tight junction complex protein), was evident within the first week after transplantation, probably as a compensatory mechanism for this TNF-α-mediated permeability disruption. Based on these findings, Oltean et al. [32] demonstrated the negative effect of IRI and loss of tight junctions on barrier function. Since most experimental animals showed no clinical manifestations of intestinal barrier disruption and had a good absorptive function and elevated citrulline levels (except in the case of severe rejection), these changes in the molecular pattern may be a part of the regular IRI and the subsequent healing process without any pathological meaning. TNF-α, zonulin and IL-6 showed maximum levels at the endpoint. This molecular-level proinflammatory state may reflect an early rejection stage because it was correlated with an “indeterminate for rejection” diagnosis in one out of the five samples studied and a “severe rejection” diagnosis in another; however, these markers are highly non-specific, and other inflammatory or infectious events may justify these findings.

Since one of the advantages of NRP and *ex vivo* perfusion is the possibility of graft intervention, further studies should address the administration of anti-TNF-α during these procedures. The use of anti-TNF-α monoclonal antibodies as induction therapy for ITx has shown beneficial effects by reducing the IRI inflammatory response in experimental rat models and humans [22, 33, 34]. In addition to IRI prevention, other strategies directly targeting the immune system, such as the administration of rabbit anti-rat thymoglobulin and fludarabine, which were recently published, could also be facilitated by NRP or *ex vivo* perfusion [35, 36].

The glucose absorption test demonstrated that the grafts’ absorptive function seems not affected. Glucose solution produced an immediate and significant increase in the glucose level from grafts’ draining veins. This elevation also occurred progressively in peripheral blood and in the veins from the native intestine, demonstrating its distribution throughout the body. This test has already been employed in other studies, sometimes with components other than glucose [17, 26]. As observed in previous studies, the use of NRP seems to be crucial to maintaining this function [25].

Regarding citrulline levels, their increase was expected since the animal received a heterotopic transplant. This increase does not have to be perfectly double, since it may have a slight decrease related to transient inflammatory events (such as the transplant itself), or to the fact that the intestine is not in use [37] or is shorter (duodenum and last part of the ileum were removed). However, a pronounced decrease seems to be related to epithelial cell apoptosis in acute cellular rejection as seen in animal 3. This is consistent with the findings of our group and others [37, 38]. It is unclear if the decreases observed in some animals could be a predictive factor for rejection since the inter-individual variability makes it difficult to clarify its value.

The difficulties and limitations of extrapolating animal research to clinical practice are well known. Our study did not include randomization since only one group was included in our proof-of-concept methodology, which is a major limitation. Previous studies have covered the differences among living donor and DCD, NRP, and *ex vivo* perfusion; therefore, repeating the same control groups was not justified ethically since this was intended to be a test to prove the feasibility of the procedure. Additionally, our study has other limitations such as the lack of more complex molecular tests and a short cold ischemia time. Nevertheless, to our knowledge, this is the first study to conduct experimental transplant of intestines from DCD with such a long and detailed follow-up, considering not only IRI but acute cellular rejection. Thus, our study could be the basis for the development of new studies to perform a proper comparison between groups in the future. A new meta-analysis would undoubtedly be warranted when more literature regarding NRP becomes available [39].

In conclusion, our experimental assay showed that NRP yielded viable intestinal grafts from DCD. Considering the feasibility, histologic, functional, and molecular results, we hypothesized that NRP could revert the deleterious IRI inherent to DCD facilitating graft viability. The preclinical model developed by us and presented in this article provided the evidence to perform the first multiviceral transplant with DCD in humans worldwide, with encouraging results in terms of graft and recipient outcome [13]. Despite recent promising results in both the clinical and experimental field, more evidence is needed to standardize the use of DCD-derived grafts in intestinal and multivisceral transplantation and reduce waiting list times.

## Data Availability

Original datasets are available upon request to the corresponding author.

## Abbreviations

CIT, cold ischemia time; Ct, threshold cycle; DCD, donation after circulatory death; ECMO, extracorporeal membrane oxygenation; EpCAM, epitelial cell adhesión molecule; fWIT, functional warm ischemic time; IEC, intestinal epithelial cells; IL, interleukin; IRI, ischemia-reperfusion injury; ITx, intestinal transplantation; LYZ, lysozyme; MLCK, myosin light chain kinase; MUC2, mucine 2; NRP, normothermic regional perfusion; PCS, Park-Chiu score; POD, postoperative day; TJP1, tight junction protein; TNF-α, tumour necrosis factor alpha; WLST, withdrawal of life support therapy.

## References

[1] Manyalich M Nelson H Delmonico FL. The Need and Opportunity for Donation After Circulatory Death Worldwide. Curr Opin Organ Transpl (2018) 23(1):136–41. 10.1097/MOT.0000000000000486 29206661

[2] Smith M Dominguez-Gil B Greer DM Manara AR Souter MJ. Organ Donation After Circulatory Death: Current Status and Future Potential. Intensive Care Med (2019) 45(3):310–21. 10.1007/s00134-019-05533-0 30725134

[3] Gozzini S Perera MT Mayer DA Mirza DF Kelly DA Muiesan P Liver Transplantation in Children Using Non-Heart-Beating Donors (NHBD). Pediatr Transpl (2010) 14(4):554–7. 10.1111/j.1399-3046.2009.01280.x 20070562

[4] Miñambres E Suberviola B Dominguez-Gil B Rodrigo E Ruiz-San Millan JC Rodríguez-San Juan JC Improving the Outcomes of Organs Obtained From Controlled Donation After Circulatory Death Donors Using Abdominal Normothermic Regional Perfusion. Am J Transpl (2017) 17(8):2165–72. 10.1111/ajt.14214 28141909

[5] Treckmann J Moers C Smits JM Gallinat A Maathuis MHJ van Kasterop-Kutz M Machine Perfusion Versus Cold Storage for Preservation of Kidneys From Expanded Criteria Donors After Brain Death. Transpl Int (2011) 24(6):548–54. 10.1111/j.1432-2277.2011.01232.x 21332580

[6] Angelico R Perera MTPR Manzia TM Parente A Grimaldi C Spada M. Donation After Circulatory Death in Paediatric Liver Transplantation: Current Status and Future Perspectives in the Machine Perfusion Era. Biomed Res Int (2018) 2018:1756069. 10.1155/2018/1756069 29744353 PMC5878911

[7] Canovai E Ceulemans LJ Peers G De Pourcq L Pijpops M Hoffman I Cost-Effectiveness of Intestinal Transplantation Compared to Parenteral Nutrition in Adults. Transplantation (2021) 105(4):897–904. 10.1097/TP.0000000000003328 32453254

[8] Lenaerts K Ceulemans LJ Hundscheid IH Grootjans J Dejong CH Olde Damink SW. New Insights in Intestinal Ischemia-Reperfusion Injury: Implications for Intestinal Transplantation. Curr Opin Organ Transpl (2013) 18(3):298–303. 10.1097/MOT.0b013e32835ef1eb 23449345

[9] Cobianchi L Zonta S Vigano J Dominioni T Ciccocioppo R Morbini P Experimental Small Bowel Transplantation From Non-Heart-Beating Donors: A Large-Animal Study. Transpl Proc (2009) 41(1):55–6. 10.1016/j.transproceed.2008.08.151 19249474

[10] Stringa P Vecchio Dezillio LE Talayero P Serradilla J Errea A Machuca M Experimental Assessment of Intestinal Damage in Controlled Donation After Circulatory Death for Visceral Transplantation. Transpl Int (2023) 36:10803. 10.3389/ti.2023.10803 36713114 PMC9878676

[11] Hartog H Brown RM Neil DA Sharif K Gupte GL Mirza DF Characterization of Ischemic Changes in Small Bowel After Normothermic Regional Perfusion: Potential to Consider Small Bowel Grafts From DCD Donors? Transplantation (2016) 100(12):e156–7. 10.1097/TP.0000000000001460 27575688

[12] Roskott AM van Haaften WT Leuvenink HG Ploeg RJ van Goor H Blokzijl T Histopathologic and Molecular Evaluation of the Organ Procurement and Transplantation Network Selection Criteria for Intestinal Graft Donation. J Surg Res (2014) 189(1):143–51. 10.1016/j.jss.2014.02.008 24655665

[13] Andres AM Encinas JL Sánchez-Galán A Rodríguez JS Estefania K Sacristan RG First Case Report of Multivisceral Transplant From a Deceased Cardiac Death Donor. Am J Transpl (2023) 23(4):577–81. 10.1016/j.ajt.2022.12.021 36725427

[14] Lao OB Healey PJ Perkins JD Reyes JD Goldin AB. Outcomes in Children With Intestinal Failure Following Listing for Intestinal Transplant. J Pediatr Surg (2010) 45(1):100–7. 10.1016/j.jpedsurg.2009.10.019 20105588 PMC2813842

[15] Organización Nacional de Trasplantes. Legislación Española en Trasplante (2022). Available from: http://www.ont.es/infesp/Paginas/LegislacionNacional.aspx (Accessed August 25, 2022).

[16] Schmittgen TD Livak KJ. Analyzing Real-Time PCR Data by the Comparative C(T) Method. Nat Protoc (2008) 3(6):1101–8. 10.1038/nprot.2008.73 18546601

[17] Stringa P Romanin D Lausada N Papa Gobbi R Zanuzzi C Martín P Gut Permeability and Glucose Absorption Are Affected at Early Stages of Graft Rejection in a Small Bowel Transplant Rat Model. Transpl Direct (2017) 3(11):e220. 10.1097/TXD.0000000000000718 PMC5682765 29184909

[18] Wang J Zhang W Wu G. Intestinal Ischemic Reperfusion Injury: Recommended Rats Model and Comprehensive Review for Protective Strategies. Biomed Pharmacother (2021) 138:111482. 10.1016/j.biopha.2021.111482 33740527

[19] Coffey JC Wanis KN Monbaliu D Gilbo N Selzner M Vachharajani N The Influence of Functional Warm Ischemia Time on DCD Liver Transplant Recipients' Outcomes. Clin Transpl (2017) 31(10). 10.1111/ctr.13068 28772351

[20] Yandza T Tauc M Saint-Paul MC Ouaissi M Gugenheim J Hébuterne X. The Pig as a Preclinical Model for Intestinal Ischemia-Reperfusion and Transplantation Studies. J Surg Res (2012) 178(2):807–19. 10.1016/j.jss.2012.07.025 22884450

[21] Timmermann W Gasser M Meyer D Kellersmann R Gassel HJ Otto C Progress in Experimental Intestinal Transplantation in Small Animal Models. Acta Gastroenterol Belg (1999) 62(2):216–20. 10427785

[22] Pech T von Websky M Ohsawa I Kitamura K Praktiknjo M Jafari A Intestinal Regeneration, Residual Function and Immunological Priming Following Rescue Therapy After Rat Small Bowel Transplantation. Am J Transpl (2012) 12(4):S9–S17. 10.1111/j.1600-6143.2012.04262.x 22974463

[23] Grant D Zhong R Hurlbut D Garcia B Chen HF Lamont D A Comparison of Heterotopic and Orthotopic Intestinal Transplantation in Rats. Transplantation (1991) 51(5):948–54. 10.1097/00007890-199105000-00003 2031277

[24] Guo M Yao D Li L Lu C Li Y Li J. Intestinal Conditioning After Cardiac Arrest: The Use of Normothermic Extracorporeal Membrane Oxygenation in the Non-Heart-Beating Animal Model. Artif Organs (2016) 40(8):738–45. 10.1111/aor.12691 27097758

[25] Hamed MO Barlow AD Dolezalova N Khosla S Sagar A Gribble FM *Ex Vivo* Normothermic Perfusion of Isolated Segmental Porcine Bowel: A Novel Functional Model of the Small Intestine. BJS Open (2021) 5(2):zrab009. 10.1093/bjsopen/zrab009 33839750 PMC8038264

[26] Guo M Lu C Li L Yao D Li Y. Normothermic Extracorporeal Membrane Oxygenation Support: Improving the Function of Intestinal Grafts Obtained From Cardiac Death Donors. Artif Organs (2020) 44(10):1098–106. 10.1111/aor.13697 32279328

[27] Søfteland JM Casselbrant A Biglarnia AR Linders J Hellström M Pesce A Intestinal Preservation Injury: A Comparison Between Rat, Porcine and Human Intestines. Int J Mol Sci (2019) 20(13):3135. 10.3390/ijms20133135 31252560 PMC6650817

[28] Chandel NS Trzyna WC McClintock DS Schumacker PT. Role of Oxidants in NF-Kappa B Activation and TNF-Alpha Gene Transcription Induced by Hypoxia and Endotoxin. J Immunol (2000) 165(2):1013–21. 10.4049/jimmunol.165.2.1013 10878378

[29] Taylor CT Dzus AL Colgan SP. Autocrine Regulation of Epithelial Permeability by Hypoxia: Role for Polarized Release of Tumor Necrosis Factor Alpha. Gastroenterology (1998) 114(4):657–68. 10.1016/s0016-5085(98)70579-7 9516386

[30] Van Welden S Selfridge AC Hindryckx P. Intestinal Hypoxia and Hypoxia-Induced Signalling as Therapeutic Targets for IBD. Nat Rev Gastroenterol Hepatol (2017) 14(10):596–611. 10.1038/nrgastro.2017.101 28853446

[31] He WQ Wang J Sheng JY Zha JM Graham WV Turner JR. Contributions of Myosin Light Chain Kinase to Regulation of Epithelial Paracellular Permeability and Mucosal Homeostasis. Int J Mol Sci (2020) 21(3):993. 10.3390/ijms21030993 32028590 PMC7037368

[32] Oltean M Joshi M Björkman E Oltean S Casselbrant A Herlenius G Intraluminal Polyethylene Glycol Stabilizes Tight Junctions and Improves Intestinal Preservation in the Rat. Am J Transpl (2012) 12(8):2044–51. 10.1111/j.1600-6143.2012.04067.x 22548829

[33] Gerlach UA Atanasov G Wallenta L Polenz D Reutzel-Selke A Kloepfel M Short-Term TNF-Alpha Inhibition Reduces Short-Term and Long-Term Inflammatory Changes Post-Ischemia/Reperfusion in Rat Intestinal Transplantation. Transplantation (2014) 97(7):732–9. 10.1097/TP.0000000000000032 24598936

[34] Gerlach UA Lachmann N Sawitzki B Arsenic R Neuhaus P Schoenemann C Clinical Relevance of the De Novo Production of Anti-HLA Antibodies Following Intestinal and Multivisceral Transplantation. Transpl Int (2014) 27(3):280–9. 10.1111/tri.12250 24279605

[35] Cicora F Stringa P Guerrieri D Vásquez D Toniolo F Roberti J Evaluation of Histological Damage of Solid Organs After Donor Preconditioning With Thymoglobulin in an Experimental Rat Model. Transpl Immunol (2013) 28(4):203–5. 10.1016/j.trim.2013.04.002 23597700

[36] Vela M Stringa P González-Navarro P Machuca M Pascual-Miguel B Mestre C Donor's Graft *Ex Vivo* T-Cell Depletion with Fludarabine Reduces Graft-Versus-Host Disease Signs and Improves Survival After Intestinal Transplantation - an Experimental Study. Transpl Int (2020) 33(10):1302–11. 10.1111/tri.13672 32526809

[37] Vecino López R Andrés Moreno AM Ramos Boluda E Martinez-Ojinaga Nodal E Hernanz Macías A Prieto Bozano G [Plasma Citrulline Concentration as a Biomarker of Intestinal Function in Short Bowel Syndrome and in Intestinal Transplant]. Pediatr (Barc) (2013) 79(4):218–23. 10.1016/j.anpedi.2013.02.007 23528708

[38] Lauro A Marino IR Matsumoto CS. Advances in Allograft Monitoring After Intestinal Transplantation. Curr Opin Organ Transpl (2016) 21(2):165–70. 10.1097/MOT.0000000000000279 26741111

[39] van de Leemkolk FEM Schurink IJ Dekkers OM Oniscu GC Alwayn IPJ Ploeg RJ Abdominal Normothermic Regional Perfusion in Donation After Circulatory Death: A Systematic Review and Critical Appraisal. Transplantation (2020) 104(9):1776–91. 10.1097/TP.0000000000003345 32541563

