# Supplementary figures and images for: Preclinical Study of DCD and Normothermic Perfusion for Visceral Transplantation
Source: Transpl Int. 2023 Sep 8;36:11518. doi: 10.3389/ti.2023.11518 (PMC10514355; doi:10.3389/ti.2023.11518)

**SUPPLEMENTAL FIGURE 1**

**Correlation between fWIT and gene expression**


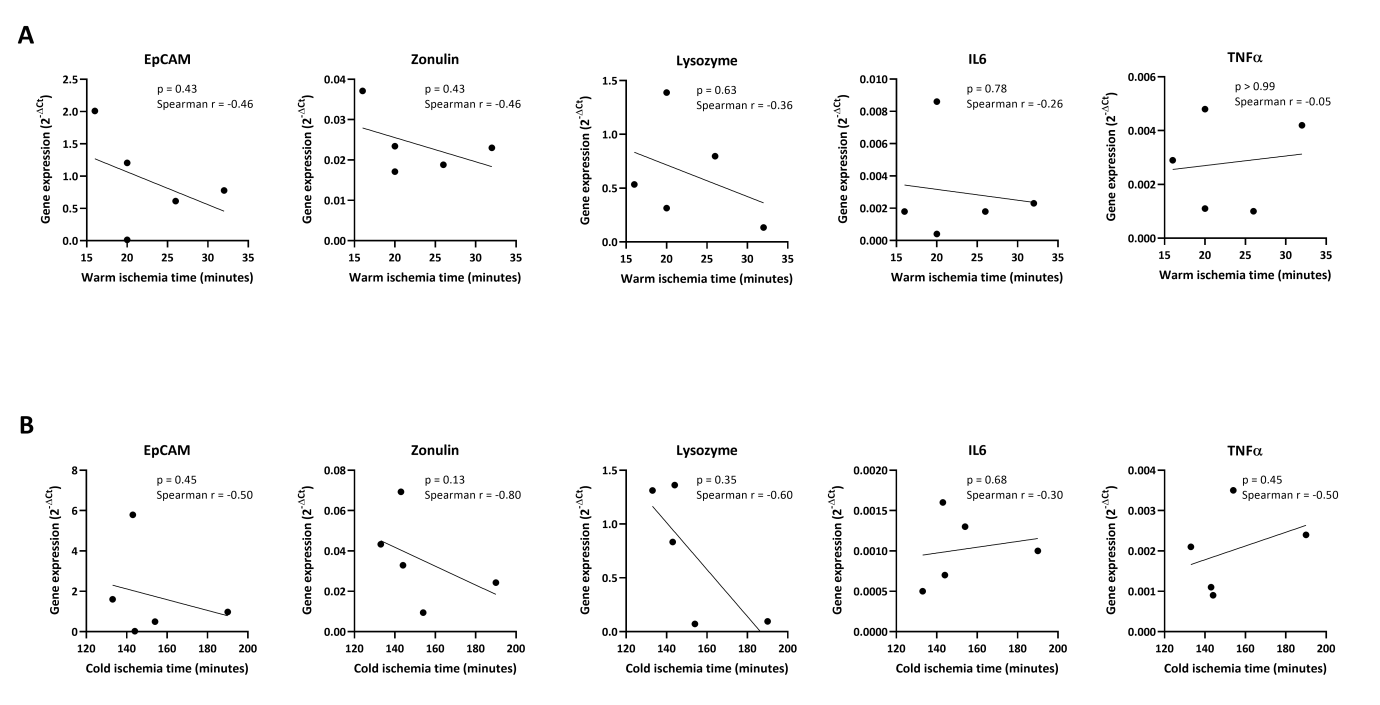

Supplement: Supplementary file 1 [file DataSheet2.doc]
