# Supplementary material for: Preclinical Study of DCD and Normothermic Perfusion for Visceral Transplantation
Source: Transpl Int. 2023 Sep 8;36:11518. doi: 10.3389/ti.2023.11518 (PMC10514355; doi:10.3389/ti.2023.11518)
Supplement: Supplementary file 2 [file DataSheet1.docx]

**SUPPLEMENTAL INFORMATION**

**Detailed procedure for NRP control and determinations during 1-h**

For arterial cannulation, a 14/16-Fr cannula was inserted (DLP™ Medtronic; Minneapolis, USA). A single-stage 20/22/24-Fr (DLP™ Medtronic; Minneapolis, USA) cannula was chosen for IVC/right atrial cannulation. Both cannulas were inserted after rapid opening of the abdominal cavity. NRP was initiated immediately after the cannulation.

NRP support for the abdominal organs was performed using a compact custom closed extracorporeal circulation circuit. The system consists of a Rotaflow™ RF-32 centrifugal pump, Bioline™-coated (Maquet Cardiopulmonary GmbH, Rastatt, Germany), and a polypropylene hollow fiber oxygenator/heat exchanger unit with Softline™ coating-heparin-free biopassive polymer- and integrated arterial filter (Quadrox-I™; Maquet Cardiopulmonary GmbH, Rastatt, Germany). The tubing set and components were albumin/heparin-coated (Bioline™ Coating, Maquet Cardiopulmonary GmbH, Rastatt, Germany) and kept to a minimum size and length.

The circuit was primed with a combination of an isotonic, buffered intravenous crystalloid solution (Viaflo Plasmalyte 148, Baxter Healthcare), and 0.2 g/kg modified fluid gelatin (Gelaspan 4%, Braun) supplemented with mannitol 10% solution (1 g/kg, Fresenius Kabi), cefazolin (15 mg/kg, Normon), methylprednisolone (20 mg/kg, Solu-Moderín, Pfizer), and sodic heparin (1000 IU/100 ml, Reig Jofré) to a total perfusate volume of 500 ± 50 mL.

During NRP, pump flow was maintained at >1.8 L/min/m^2^ to ensure effective therapy (>60% of the intended full flow for a high-demand perfusion procedure). Rectal temperature was maintained at >36 °C. An oxygen/air mixture was delivered to the oxygenator on sweep gas levels adjusted to reach PaCO_2_ between 35 and 45 mmHg and SaO_2_ > 98%.

Blood was sampled at 5 min and every 30 min to determine arterial/venous biochemical and gasometrical data to adjust the functional parameters of the NRP (Radiometer ABL 90). Sodium bicarbonate (8.4% solution) was added to the perfusate to maintain physiological pH if metabolic acidosis persisted despite optimal perfusion management. Calcium correction was also applied if the values persisted below 1 mmol/L. Insulin (Actrapid™ Nova Nordisk) was administered if glycemia > 280 mg/dL was noted at 30 min of NRP. Unfractionated heparin was added continuously to obtain an activated clotting time > 250 s, measured at the beginning of perfusion and every 20 min by a Hemochron Signature Elite™ Whole Blood Microcoagulation System. During the procedure, targeted flow was reached through volume correction (Plasmalyte 148) and phenylephrine boluses (200 µg/ml).

The following oxygen-derived parameters were calculated at NRP T5, T30, and T60:

-CaO_2_: Arterial oxygen content (mL [100 mL]^-1^) = hemoglobin (g·dL^-1^) × 1.34 (mL O_2_·g^-1^ of hemoglobin) × SaO_2_ (%) + 0.003 × PaO_2_ (mmHg), where SaO_2_ is the arterial oxygen saturation and PaO_2_ is the arterial oxygen partial pressure.

· CvO_2_: Venous oxygen content (mL [100 mL]^-1^) = hemoglobin (g·dl^-1^) × 1.34 (mL O_2_·g^-1^ of hemoglobin) × SvO_2_ (%) + 0.003 × PvO_2_ (mmHg), where SvO_2_ is the venous oxygen saturation and PaO_2_ is the venous oxygen partial pressure.

· Indexed O_2_ delivery: D_i_O_2_ (mL·min^-1^·m^-2^) = 10 × pump flow index (L·min^-1^·m^-2^) × CaO_2_

· Indexed O_2_ consumption: V_i_O_2_ (mL·min^-1^·m^-2^) = 10 × pump flow index (L·min^-1^· m^-2^) × CvO_2_

· O_2_ extraction ratio: ERO_2_ (%) = VO_2_ (mL·min^-1^)/DO_2_ (mL·min^-1^) × 100

Venous-arterial CO_2_ to arterial-venous O_2_ content difference ratio (∆PCO_2_/C_a-v_O_2_) was also calculated.
